# Global Emerging Pathogens, Poverty and Vulnerability: An Ethical Analysis

**DOI:** 10.1007/978-3-030-17474-3_18

**Published:** 2019-03-20

**Authors:** Mbih Jerome Tosam, J. Radeino Ambe, Primus Che Chi

**Affiliations:** 1 Department of Philosophy, University of Yaounde 1, Yaounde, Cameroon; 2 Global Emerging Pathogen Treatment Consortium (GET) Consortium, Lagos, Nigeria; 3 Global Emerging Pathogens Treatment Consortium, Lagos, Nigeria; 4 Department of Medicine, University of Cape Town, Cape Town, South Africa; 5 Department of Philosophy, University of Bamenda, Bamenda, Cameroon; 6 Cameroon Bioethics Initiative, Yaounde, Cameroon; 7 Department of Public Health, School of Nursing and Health Sciences, Capella University, Minneapolis, MN USA; 8 The Global Emerging Pathogens Treatment Initiative, Lagos, Nigeria; 9 KEMRI-Wellcome Trust Research Programme, Kilifi, Kenya

## Abstract

In the last few decades, the world has witnessed the emergence and re-emergence of new and old infectious diseases. Emerging infectious diseases (EIDs) have the capacity to spread rapidly from one region of the world to another, within a very short time, due to world travel and increased global interdependence. The impact of this varies from one region to another. Resource poor countries suffer the most due to an already high disease burden, poor infrastructures, lack of clean, potable water and sanitation, as well as an acute shortage of qualified health personnel to manage, control and contain the crisis/spread. Poor and marginalized communities are the most vulnerable because infectious diseases cause not only suffering and death, but also severe economic hardship. The outbreak of HIV/AIDS, tuberculosis and Ebola Virus Disease (EVD) in the developing world has shown the extent to which economic and social conditions can affect vulnerable populations. These socio-economic, cultural and environmental conditions accelerate the spread of, and exacerbate the negative impact of emerging pathogens. This chapter will undertake an analysis of the trend in global emerging pathogens, their economic impact, the global vulnerability status and ethical implications.

## Introduction

Emerging infectious diseases (EIDs) are “*newly identified* or known infectious diseases that have either expanded in geographic range or increased in infection prevalence over the previous two decades” (Liu and Yu 2017, 12). Some examples are the recent Ebola Virus Disease (EVD) in the West African countries of Guinea, Sierra Leone and Liberia, the Middle East Respiratory Syndrome Coronavirus (MERS-CoV) that has had large outbreaks in Saudi Arabia, United Arab Emirates, and the Republic of Korea, and Zika Virus Disease (ZVD) that affected many countries in the Americas and beyond.

Emerging infectious diseases pose serious public health concerns and cause major socio-economic consequences in affected persons and populations. The effects and impact of EIDs on individual and population health may vary from one individual to another and from one society to another. Globally, the distribution of EIDs vary between countries; as well as within each country. A strong health system is critical in effectively combating EIDs through the establishment of strong infection prevention and control programmes. Strong and resilient health systems better tackle these diseases and prevent them from assuming epidemic and pandemic levels.

Socio-economic, cultural and environmental conditions play a fundamental role in the emergence, spread and control/management of EIDs. In poor communities, a large part of the population live in overcrowded and squalid conditions. In these communities, mostly in the major cities of developing countries, there is a lack of clean drinking water, poor hygiene and sanitation. This environment creates opportunities for waterborne diseases and different forms of pollution. It is in the slums of the main cities of developing countries that most EIDs begin and spread and it is also in such areas of the cities that the greatest number of deaths are usually recorded. Infectious diseases like Ebola, HIV, TB, usually spread easily and widely from poor communities in emerging cities and through health workers who serve such communities. Hence, poverty creates a favourable condition for the spread of infectious diseases and makes it difficult for affected people to get adequate access to prevention and care (WHO 2012a, b). The journal of *Infectious Disease of Poverty was* launched in 2012 with the principal aim of fostering “*interdisciplinary and transdisciplinary research that explicitly highlights the intersection of poverty and other ecological factors with disease”* (Xia et al. 2013).

In this chapter, we critically examine the socio-economic and environmental factors that influence the emergence and spread of EIDs and discuss the ethical issues that arise from the global response and management of EIDs.

## Trends and Distribution

**Fig. 18.1:**
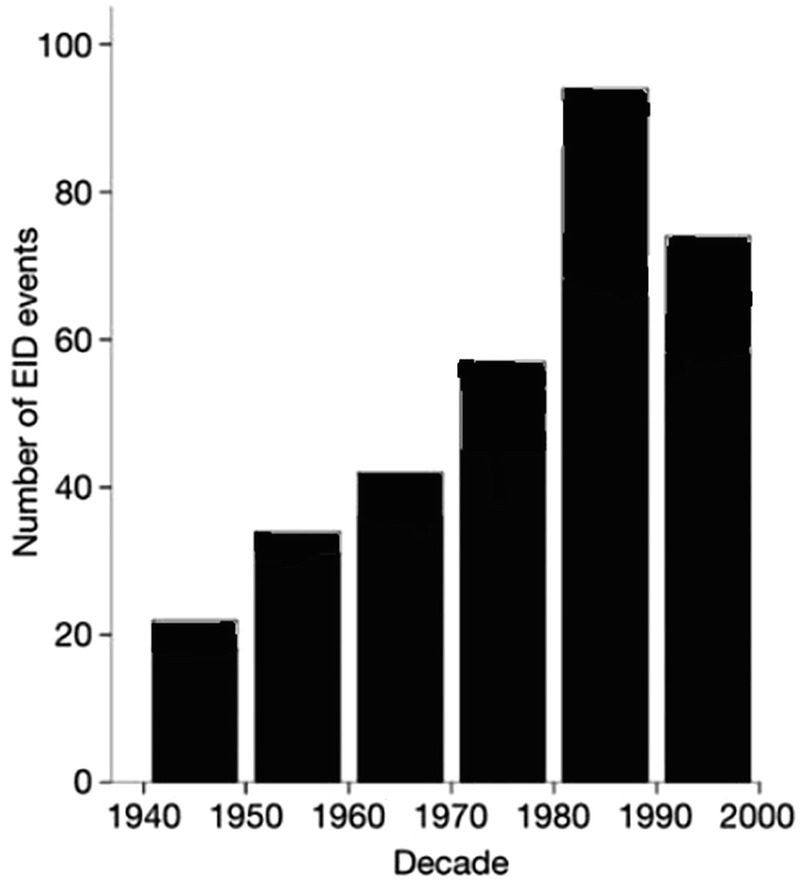
Distribution of EID events from 1940 to 2000. (Adapted from Jones et al. 2009)

Globally, the trend in the outbreak of EIDs has been increasing. Jones et al. (2009) analysed the trend of EIDs events from 1940 to 2000, identifying a total of 335 EID events. They found that during this period, the number of EID events ranged from just over 20 between 1940 and 1950 to close to 80 events between 1990 and 2000, with a peak of close to 100 events between 1980 and 1990 as shown on Fig. 18.1. This peak was associated with the HIV/AIDS pandemic.

This increasing trend in EID mirrors the overall global trend in all human infectious diseases. For instance, Smith et al. (2014) found that within a 33-year dataset (1980–2013), 12,102 outbreaks of 215 human infectious diseases were reported, with more than 44 million cases occurring in 219 nations.

**Fig. 18.2:**
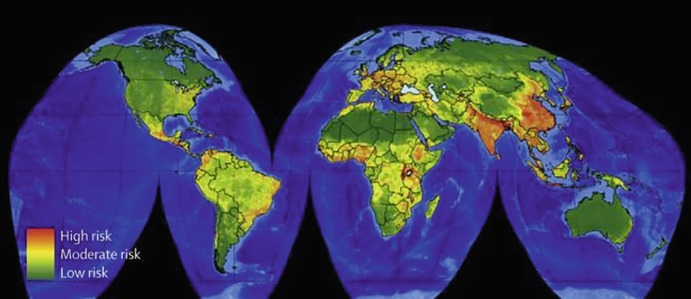
Global distribution of risk of EIDs originating from wildlife. (Source: Morse et al. 2012)

Emerging infectious diseases are distributed all over the world, although their rate of emergence and spread varies from one setting to another. Farmer (1996) observed that their emergence and spread appear to be high in areas with huge social inequalities. Additionally, EIDs are more common in areas rich in wildlife and zoonotic pathogens from wildlife while vector-borne pathogens are more concentrated in lower latitudes, such as tropical Africa, Latin America and Asia (Jones et al. 2009). Also, specific EIDs appear to be common within certain geographical regions. For example, EVD has mainly been in sub-Saharan Africa while the ZVD has been largely limited to the Americas, situations which might be associated with the geographical location of the vectors that spread the disease. Different authors have developed global maps highlighting the areas where EIDs have originated or are most likely to originate. These areas have been described as EID ‘hotpots’. One of those maps has been developed by Morse and colleagues, and illustrates the risk of emergence of infectious diseases originating from wildlife as shown in Fig. 18.2 below (Morse et al. 2012).

## Factors Affecting the Emergence of Infectious Diseases

The emergence of these diseases is driven by socio-economic, environmental and ecological factors (Jones et al. 2009). The major factors include ecological changes (including those due to economic development and land use), human demographics, behaviour, international travel and commerce, technology and industry, microbial adaptation and change, and breakdown in public health or control measures (Morse 2004). Examples of specific factors affecting infectious disease emergence is shown on Table 18.1. There is a direct correlation between poverty and the emergence of infectious diseases which can be seen through different factors. For instance, the ecological footprint left by humankind is evident by the direct impact on the land, air and water of a specific area or region, used to sustain the depletion of natural resources for an individual or a community.

**Table 18.1:** Factors associated with the emergence of infectious diseases and their relationship with poverty^a^

Factors	Examples of specific factors	Relationship to poverty
Ecological changes (including those due to economic development and land use)	Agriculture, dams, changes in water ecosystems, deforestation/reforestation, flood/drought, famine, climate change	Due to poverty, humans may move into new areas (eg. In search of food or shelter - forest clearing for agriculture, wildlife hunting) where there is a higher likelihood for infection. For example forest clearing and wildlife hunting exposes humans more to wildlife that may serve as reservoirs for these infections
Human demographics, behaviour	Societal events: population migration (movement from rural areas to cities), war or civil conflict, economic impoverishment, urban decay, factors in human behaviour such as the commercial sex trade, intravenous drug use, outdoor recreation, use of childcare facilities and other high-density settings	Growing urbanisation or conflicts, may force humans, especially the poor into behaviours (risky sexual habits) that may increase the likelihood of mergence and spread of infectious diseases.
International travel and commerce	Worldwide movement of goods and people, air travel	Increasing international travels to major international cities may increase the cost of living and force poorer individuals to areas where there is increased contact with EID vectors or engage in risky behaviours that may lead to infectious disease emergence/ re-emergence
Technology and industry	Food production and processing: globalisation of food supplies, changes in food processing and packaging	Poverty may cause poorer people to sell their organs which may decrease their immune-competence and enhance the re-emergence of infectious disease
	Health care: new medical devices, organ or tissue transplantation, drugs causing immunosuppression, widespread use of antibiotics	
Microbial adaptation and change	Microbial evolution, response to selection in the environment	Poorer people are more likely to be engage in self-prescription of antibiotics due to the cost associated with seeing a qualified health professional. Such practices might increase the re-emergence of EIDs associated with antibiotics resistance.
Breakdown in public health or control measures	Curtailment or reduction in disease prevention programmes; lack of or inadequate sanitation and vector control measures	A cutback in public spending on disease and prevention programmes may be more severe in rural areas where the poorer and often less assertive people live compared to major urban centres where the high economic class lives.

^**a**^Adapted from Morse (2004)

**Fig. 18.3:**
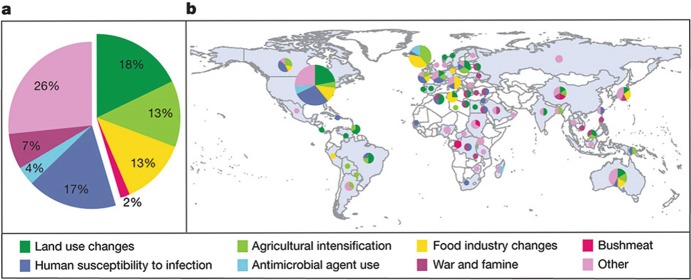
Drivers and locations of emergence events for zoonotic infectious diseases in humans from 1940 to 2005. (Source: Keesing et al. 2010)

Among the EIDs of zoonotic origin that make up more than 60% of all EIDs, evidence suggests that between 1940 and 2005 their emergence was largely attributed to changes in land use (18%), human susceptibility to infection (17%), intensification of agricultural practices (13%), and changes in the food industry (13%) (Keesing et al. 2010). A combination of ‘other’ factors (international travel and commerce, changes in human demographics and behaviour, changes in the medical industry, climate and weather, breakdown of public health measures, and unspecified causes) accounted for 26% of the drivers. Figure 18.3 shows the global percentage of emergence events caused by each driver (a) and the countries in which the emergence events took place, and the drivers of emergence (b) from 1940 to 2005 (Keesing et al. 2010).

This study found that a decrease in the diversity ecosystem that is associated with changes in land use, changes in agricultural and other food production practices such as wildlife hunting, which has led to increasing contacts between humans and other animals has facilitated the emergence of infectious diseases of zoonotic origins (Keesing et al. 2010).

## Poverty and the Emergence of Pathogens

There is a close nexus between poverty and infectious diseases. Poverty has provoked a wave of rural urban migration of people in search of new opportunities, which has led to population explosion in the major cities of most developing countries. The result of this has been the growth of slums and expanding cities with “new opportunities for infectious diseases to flourish and spread” (Eisenstein 2016). The concentration of people in squalid conditions leads to waterborne diseases and different forms of pollution. Also, the magnitude and pace of the spread of infectious diseases is usually influenced by overcrowding in tandem with poor hygiene and sanitation. For example, it is reported that the recent Ebola outbreak in West Africa spread widely and rapidly in densely populated and highly mobile, urban slums in cities like Monrovia and Conakry (Eisenstein 2016). It was particularly among poor people in the slums, who lacked basic hygiene and sanitation infrastructure in the cities, that there was a serious outbreak. The UN-Habitat estimates that 863 million people- one-third of the developing world’s urban population live in slums. Most slums do not have access to safe and clean drinking water and where there exist, it is usually easily open to contamination. These conditions are favourable for the spread of diseases such as cholera, a bacterial diarrhoea and typhoid infections which are transmitted through food and water. For instance, cholera outbreaks were almost an annual occurrence in the northern part of Cameroon during the past 10 years. What accounts for this is extreme water shortage and climate variability, poor sanitation, poor drainage systems with no toilet facilities, in some areas, so that during the rainy season, water carries all human and animal waste into the main sources of water for household use. In some parts of this region, humans and animals depend on the same source of water (Nfor 2014). This exposes the people to waterborne and food related diseases like cholera and typhoid.

Moreover, the “urban poor” usually travel far and wide in search of work, greatly increasing the areas that could be affected by the virus and making contact tracing very difficult (Eisenstein 2016). In slums, there are small ponds, abandoned vehicles, tyres and plastic waste which serve as ideal habitat for the insects that spread dengue and yellow fever as well as malaria. Improvement in living conditions-less crowding, fewer animals and higher quality homes and education may help reduce the possibility of people contracting and spreading infectious diseases.

### Deforestation, Global Warming and Climate Change

In addition to the aforementioned factors, high burden of disease, fragile health systems and socio-economic disparity aids in the proliferation of disease vectors and increases vulnerability which can be seen in the patterns of global warming on the African continent. The earth has an ecological system that is comprised of biospheres that are interconnected and rely on each other. Once that is interrupted, for instance, with migration, this disrupts the balance of the biospheres and adds to the burden of this disruption and environmental decay (Abayomi and Cowan 2014). Other factors are increased temperatures, rising sea levels, and increased air pollution. These adverse climatic conditions cause food and waterborne diseases as well as regular ruthless natural disasters (Tosam and Mbih 2015).

Moreover, circumstantial evidence shows that deforestation may have played a role in the West African Ebola Epidemic of 2014/2015. The index case for the West African Ebola Virus Disease Outbreak, lived in a district known as Maliandou, in a village called Guéckédou, in Guinea- Conakry (Marí Saéz 2014). This area is known as the Forest Region, however only approximately 20% of the trees are still standing. The area has lost most of the vegetation due to the mining of iron ore, bauxite, gold and aluminium (Marí Saéz 2014). Wild animals have lost their ecosystems and many use the roofs of thatched huts to nest, living in closer proximity to the villagers. The few remaining trees, are colonized by bats and other animals. It is thought that the insectivorous bats in the hollowed-out tree, in the yard of the index case, may have been the reservoir for the Ebola Virus, Zaire strain (EBOV). Research shows that this particular species (*Mops condylurus*) are able to survive infection by EBOV (Marí Saéz 2014). Amongst the first twelve EVD cases, none were hunters and the index case, was a toddler which led epidemiologists to believe that domestic spaces were in danger for the spread of the disease. Clearly, close living proximity between the bats and the people of the village provides circumstantial evidence of the mode of transmission of this disease, given the issues with deforestation (Borchert et al. 2015).

## Ethical Implications in the Context of Poverty

While EIDs have contributed to exacerbating global health inequalities, inequalities in socio-economic conditions globally have arguably also contributed to the emergence and re-emergence of infectious diseases. As has been demonstrated in the earlier sections, developing regions (mostly low and middle-income countries) and impoverished communities and people tend to bear the brunt of EIDs and it negative impacts. Considering the prevailing situation, it would be expected that any efforts in managing the emergence and re-emergence of infectious disease would place more attention and resources in addressing the root causes, especially in developing and low-resource countries. Additionally, one will expect that countries and regions with relatively lower capacity to detect these diseases should receive more attention as the pace with which disease outbreaks are recognized is critical for establishing effective control efforts (Kluberg et al. 2016). However, existing evidence suggests that global resources devoted to countering the emergence of infectious diseases are poorly allocated, with the majority of the scientific and surveillance effort focused on countries from where the next important EID is least likely to originate (Jones et al. 2009). This raises serious ethical issues as it would be expected that for a more effective global response to the prevention and control of EIDs, more resources should be channeled to regions and countries with the greatest risk of experiencing the emergence of these diseases.

Due to our shared elements of vulnerability, there is an urgent need for international cooperative endeavours to promote and preserve health since EIDs know no geographical and economic borders. In the past, vulnerability to EIDs and other health challenges was defined by geographic location and economic factors, but because of the high level of international interactions and movement of persons and goods across borders, this is no longer the case. And since those in affluent countries benefited from the accident of geography and climate as well as efficient health infrastructures which protects them from many threats to EIDs, it is difficult for many to identify with vulnerable people in poor countries. On the contrary, poor people, as a result of their geographical location (mostly in tropical regions) with harsh climatic and economic situations, weak and inefficient health infrastructures; lack access to the health goods needed to prevent them from EIDs (West-Oram and Bux 2016, 1). However, because of globalization and climate change, the tides are changing and vulnerability is being redefined. Today, protection or exposure to EIDs and other tropical diseases is no longer determined by geographical location or economic situation; “all persons are increasingly united in their vulnerability to emerging threats” (West-Oram and Bux 2016, 1). There is need for global cooperation and solidarity between the affluent and poor nations. This can only be effective if both parties identify with each other and acknowledge their shared vulnerability. For example, with the ease in international air travels, an EID in sub-Saharan Africa (sSA) or Asia can reach Europe or the USA within a couple of hours.

The recent EVD and Zika virus pandemics have firmly revealed the extent of global vulnerability and response to EIDs. While EVD-infected expatriate health personnel were flown to their countries for treatment, local EVD-infected health personnel were not accorded such treatment. This attitude was motivated by the failure to recognize the similarity between citizens in rich and poor countries which emerging health threats has exposed us to in our increasingly interconnected interdependent world. It further reflects the discrepancy that existed in international health in the past which does not longer hold today. This calls for a paradigm shift from the charity-based approach to a solidaristic one (West-Oram and Bux 2016, 7). An approach that acknowledges our global interdependence and shared vulnerability to global health threats such as EIDs; and recognizes that if a neighbour’s home is on fire, efforts must be made by all to put off the fire, otherwise it may spread and consume more homes (including ours) that may even be further away from the initial home on fire! In fact, the WHO has proposed solidarity as one of the key ethical principles in the management of infectious disease outbreaks globally (WHO 2016). This principle justifies engagement in collective action in the face of common threats such as EIDs, while supporting efforts to overcome inequalities that undermine the well-being of minorities and groups suffering from discrimination. One potential application of this principle globally is the provision of financial, technical, and scientific assistance by high income and developed countries to low-income and impoverished countries to boost their capacities to prevent and manage ongoing and future EIDs. This is in fact one of the obligations of governments and the international community in the WHO ‘Guidance for managing ethical issues in infectious disease outbreaks’ (WHO 2016).

Therefore, ensuring support for low resource and poorer countries in the prevention and management of EIDs through global solidarity, global health will also be enhanced by reducing the risk of EIDs spreading to other countries. This can be achieved through strengthening LMICs’ capacities to adopt and effectively implement the International Health Regulations, a legally binding instrument of international law that aims among others to assist countries to work together to save lives and livelihoods endangered by the international spread of diseases and other health risks.

It is expected that affluent countries have a moral responsibility to support or sponsor research for neglected tropical diseases as well as emerging infectious diseases, and the countries and regions most affected need to take the lead in responding to and in contributing resources to support affected persons, not only as a duty, but on the basis of rational self-interest. Moreover, solidarity does not require that only the rich should identify with the poor; it involves identifying with all persons be they rich or poor. For example, during EVD outbreak in West Africa in 2013–2016, most African countries and the African Union were sluggish in taking the lead in the fight against the disease (Metz 2017). By the time Western countries had pledged 1 billion US dollars, African countries had barely managed to raise $700.000.Cuba, alone, had sent more than 400 health workers whilst the AU had just started deploying only 100 medical personnel (Metz 2017). Developing countries are usually ill-prepared to monitor, prevent and manage the outbreak of these diseases.

## Conclusion

In this chapter, we have shown that there is an inextricable link between socio-economic, cultural and environmental conditions and the emergence or re-emergence of EIDs. EIDs have contributed in exacerbating global health inequalities as most areas where EIDs are common are also areas that experience lack of access to basic life-saving and preventive medicines. For any fight against these diseases to be successful, mechanisms must be put in place to redress these determinants as well as bring together the intellectual, financial and health resources of the world for all, especially people from low and middle-income countries where the local capacity to appropriately manage EIDs is relatively weak. This is because in our interconnected and interdependent world, no individual, group or nation is insulated from the threats of EIDs. It is important that rich countries play a fundamental role in dedicating resources and increasing funding for research in capacity-building and drugs for EIDs in developing countries, not only because their own populations are also vulnerable to EIDs, but also for the sake of global solidarity. Also, the countries where EIDs are more likely to occur and those whose capacity to effectively manage EIDs is weak, must also play a leading role in addressing the socio-economic, cultural and environmental conditions which facilitate the emergence and spread of infectious diseases.
